# Decision-Making About Respiratory Syncytial Virus Immunization for Infants: Qualitative Interview Study

**DOI:** 10.2196/100656

**Published:** 2026-11-09

**Authors:** Leslie C M Johnson, Ilse Campos, Gabriella Salazar, Lavanya Vasudevan

**Affiliations:** 1 Department of Family and Preventive Medicine, School of Medicine, Emory University, 1518 Clifton Rd. NE, Atlanta, GA, 30322, United States, 1 (404) 727-5630; 2 Hubert Department of Global Health, Rollins School of Public Health, Emory University, Atlanta, GA, United States

**Keywords:** respiratory syncytial virus, immunization, pregnancy, infants, monoclonal antibody shot

## Abstract

**Background:**

Respiratory syncytial virus (RSV) is the leading cause of infant hospitalization in the United States. Although maternal RSV vaccination and infant RSV monoclonal antibody products are recommended to protect infants during their first RSV season, uptake has remained modest. Understanding how pregnant individuals make decisions about these products is critical for informing strategies that support timely immunization.

**Objective:**

The study aimed to examine the behavioral and social drivers shaping decisions regarding RSV vaccination during pregnancy and infant RSV monoclonal antibody immunization among pregnant individuals in the United States.

**Methods:**

We conducted an exploratory qualitative study from a relativist epistemological perspective, using semistructured interviews with 12 pregnant and postpartum adults recruited across the United States between October 2024 and May 2025. Participants were purposively sampled to include individuals with positive, uncertain, and negative intentions toward RSV vaccination. Interviews were guided by the World Health Organization’s behavioral and social drivers of vaccination framework and analyzed using a framework analysis approach. Screening survey data were summarized descriptively to characterize participant immunization intentions and contextualize the qualitative findings.

**Results:**

RSV immunization decisions reflected interacting behavioral, emotional, social, and practical influences rather than attitudes alone. Participants with positive vaccination intentions described greater trust in medical expertise, confidence in vaccination, and personal or professional experience with RSV illness. Those with unsure or negative intentions emphasized the novelty of RSV vaccines, concerns about safety and long-term effects, and greater reliance on clinician recommendations and peer experiences when making decisions. Across intention groups, participants expressed a preference for receiving protection during pregnancy rather than after birth but reported uncertainty related to vaccine availability, timing within the 32- to 36-week eligibility window, and inconsistent integration into prenatal care. Participants consistently identified trusted clinician communication, opportunities for early discussion, and access to clear educational materials as important influences on decision-making.

**Conclusions:**

Decisions regarding RSV immunization during pregnancy are shaped by interconnected motivational, interpersonal, and structural factors. Integrating education on RSV and RSV immunization options earlier in prenatal care, strengthening clinician recommendations, and reducing logistical barriers to maternal vaccination may improve informed decision-making and timely uptake of infant RSV protection.

## Introduction

Parental decisions about childhood immunizations involve a complex interplay of factors, including perceptions on vaccine efficacy, safety, and timing. Although childhood immunization is primarily administered after birth, protection against some diseases can be conferred to infants proactively in utero or through breastmilk following maternal immunization. Recent additions to the US immunization schedule include products that protect against respiratory syncytial virus (RSV), the leading cause of infant hospitalization nationwide. There are an estimated 58,000 to 80,000 RSV-associated hospitalizations and 100 to 300 deaths annually among children aged <5 years in the United States [1]. Infants face the highest RSV risk in the first months of life, underscoring the need for early protection [1,2].

RSV protection for infants can be achieved in 1 of 2 ways: through a single maternal RSV vaccine dose administered at 32 to 36 weeks of gestation or through a single dose of a long-acting monoclonal antibody (nirsevimab or clesrovimab) administered to infants aged <8 months entering their first RSV season, as well as certain older high-risk infants [2-4]. Surveillance from the 2024-2025 season showed substantially lower hospitalization rates among infants aged ≤7 months compared to those observed during prepandemic years, coinciding with the availability of these interventions [4]. Together, these findings indicate that immunization strategies can meaningfully reduce severe RSV outcomes in the first months of life.

Despite these benefits, early RSV immunization coverage has been modest and uneven. During the 2023-2024 season, only 29% of infants across 33 states and the District of Columbia were protected by either intervention, with coverage ranging from 11% to 53%; among infants receiving the infant RSV monoclonal antibody (nirsevimab), just 38% received the dose within the first week of life [5]. Rollout challenges included supply constraints and coordination between prenatal and newborn care settings [6,7]. Although families generally value RSV protection, preferences for maternal vaccination and the infant RSV monoclonal antibody vary based on perceived disease risk, information about safety and efficacy, and guidance from trusted clinicians [8-10], reflecting established behavioral and social drivers (BeSD) of vaccine uptake [11]. The aim of this qualitative study was to examine BeSD of maternal RSV vaccination and infant RSV monoclonal antibody uptake, with a focus on information seeking and interpretation. The findings are expected to inform strategies to support RSV immunization within prenatal and newborn care systems.

## Methods

### Study Design

We conducted an exploratory qualitative study from a relativist epistemological perspective using semistructured interviews with US adults who self-reported being pregnant at the time of recruitment. Interviews sought to capture a contextualized understanding of participants’ attitudes and beliefs toward maternal vaccination and to examine how these perspectives shaped decision-making about RSV immunization products.

### Conceptual Framework

We used the World Health Organization’s (WHO) BeSD of vaccination framework to guide data collection and analysis in this study [11]. The BeSD framework consists of four domains: (1) thinking and feeling; (2) social processes; (3) motivations; and (4) practical issues. Recent work has adapted and tested the BeSD tools to examine maternal vaccine acceptance and uptake [12]; however, no studies have yet applied the framework to understand confidence and decision-making about RSV immunization products during pregnancy.

### Participants and Sampling

Participants were purposively recruited using multiple strategies to maximize the reach among pregnant individuals. Recruitment included physical flyers posted in libraries and clinics, virtual flyers shared in daycare groups, searches on the ResearchMatch database (Vanderbilt University) using pregnancy as a health status criterion [13], and social media advertising. Eligibility criteria required participants to be pregnant at the time of recruitment, be aged ≥18 years, reside in the United States, have access to a cell phone or laptop, and be proficient in English.

### Data Collection

Data collection occurred between October 2024 and May 2025. During screening, participants confirmed eligibility, provided demographic information, indicated whether they had received or planned to receive the RSV vaccine during pregnancy, and reported their likelihood of accepting the infant RSV monoclonal antibody (hereafter referred to as the antibody shot) after birth. Immunization intention was used to stratify participants and was not an eligibility criterion. Accordingly, the sample included participants with positive, uncertain, and negative intention regarding immunization. Screening data were collected and managed using REDCap (Vanderbilt University) tools. REDCap is a secure, web-based software platform designed to support data capture for research studies using an intuitive interface, validated data capture, audit trails to track data manipulation, and automated data export [14].

Individuals recruited outside of ResearchMatch contacted the study team via phone or email and were screened for eligibility by a research assistant, who scheduled interviews for eligible participants who provided informed consent. A semistructured interview guide was developed using the BeSD framework to examine barriers and facilitators to vaccination during pregnancy, with a focus on RSV immunization products [11]. Questions were developed by the study team to explore the BeSD domains of thinking and feeling, motivations, social processes, and practical issues. Additional questions were included to examine participants’ decision-making processes regarding RSV immunization, including how they weighed maternal RSV vaccination and infant RSV monoclonal antibody immunization options. As clesrovimab was not yet approved in the United States at the time of the study, interviews focused on nirsevimab. Probes were used to elicit contextual, individual, interpersonal, and product-related determinants of immunization intention and behavior. The interview guide was pilot-tested before implementation. Because only minor refinements were identified (ie, the sequencing of questions and the addition of probes) and no substantive changes were made, data from the pilot interview were included in the final analysis. The full interview guide can be found in Multimedia Appendix 1.

Interviews were conducted remotely via Zoom (Zoom Communications Inc) and audio-recorded with participant permission. All interviews were conducted by a female researcher with doctoral training in qualitative methods, with support from a female research staff member with master’s-level training who served as a notetaker. Audio files and transcripts were stored on a password-protected shared drive accessible only to study staff. Interviews averaged 43 minutes.

### Data Analysis

Data collection, preparation, and analysis were conducted in parallel on an ongoing basis to ensure that the data were sufficient in richness and relevance to the study aims. Interview transcripts were transcribed using Zoom, reviewed for accuracy, and deidentified by the study team.

A qualitative framework analysis was conducted using the BeSD domains. The initial codebook was adapted from the WHO and United Nations Children’s Fund BeSD of Vaccination guide [11] and combined deductive framework-based codes with inductive codes developed through the researchers’ interpretation of participant accounts within each domain. Two master’s-trained research assistants coded transcripts in MAXQDA (version 24; VERBI Software GmbH) [15]. Two transcripts were initially coded independently to identify differences in the interpretation and application of codes. These interpretations were discussed, with input from a senior qualitative researcher, and the codebook was refined to clarify conceptual boundaries. The initial transcripts were then recoded using the refined codebook before independent coding of the remaining transcripts.

Coding and interpretation proceeded iteratively, with previously coded transcripts revisited as the analytic framework was refined so that the developing interpretations could be considered across the dataset. By the tenth interview, the analytic framework was sufficiently developed to support interpretation across the BeSD domains. Two additional interviews contributed further contextual detail but did not substantially alter the analytic framework. Considering the specificity of the sample, the use of an established conceptual framework, and the quality of the interview data, the research team determined that the dataset provided sufficient information power to address the study aims [16].

Following coding, 2 members of the study team reviewed code trees organized by BeSD domain and developed thick descriptive summaries that synthesized participants’ accounts within and across domains. These summaries formed the basis for identifying patterns and contrasts relevant to RSV immunization decision-making and selecting illustrative quotations within each framework domain. Quantitative screening data exported from REDCap were cleaned and analyzed in Microsoft Excel (version 2502) to characterize participants and generate transcript-level variables indicating immunization intention. These variables were used after the qualitative analysis to examine how the interpreted patterns varied among participants with positive intentions compared with those with unsure or negative intentions. The resulting analytic patterns were organized within the BeSD domains and provided the basis for the findings presented below. To enhance trustworthiness and credibility, the thick descriptive summaries were reviewed and discussed by the research team to ensure that interpretations remained grounded in participants’ accounts, reflected the range and nuance of perspectives expressed, and were supported by representative quotations from the data. These discussions encouraged reflexive consideration of alternative interpretations before finalizing the descriptive summaries.

The study is reported in accordance with the COREQ (Consolidated Criteria for Reporting Qualitative Research) checklist [17].

### Reflexivity Statement

Consistent with a relativist epistemological perspective, we recognize that the findings are interpretations developed through interactions among participants, researchers, the BeSD framework, and the analytic process rather than objective representations inherent in the data. The first author, who conducted the qualitative interviews and is also a parent, brought both methodological expertise and personal experience with parental health decision-making to the research process. This perspective may have shaped how participant accounts were explored during interviews and interpreted during analysis. The research team also included investigators with training and experience in qualitative and mixed methods research, implementation science, global public health, maternal vaccination, and maternal and child health. The research assistants responsible for coding were not parents, providing perspectives distinct from those of the first author. During analysis, differences in interpretation were discussed among the research team, with attention to how personal experiences and disciplinary perspectives could shape analytic decisions. The team also considered alternative interpretations and participant accounts that complicated or challenged the developing interpretations before finalizing the findings.

### Ethical Considerations

This study (number 00008274) was reviewed by the Emory University Institutional Review Board and determined to be exempt from human subjects research requirements. All participants provided electronic informed consent prior to participation. Interview transcripts were deidentified before analysis, and audio recordings and study materials were stored on password-protected institutional servers accessible only to approved study personnel. Participants were provided with a US $50 Visa electronic gift card as compensation for their time.

## Results

### Participant Characteristics

A total of 12 interviews were conducted with English-speaking participants who were pregnant at enrollment. Two-thirds (n=8) were in their second trimester, and none was beyond 36 weeks of gestation. The average age of participants was 32.8 (SD 2.56) years. Most participants self-identified as White (n=10), including 2 who also identified as Hispanic or Latino and Middle Eastern. One participant identified as Black or African American, and 1 identified as Asian. Participants were geographically distributed across the South (n=8) and the Midwest or West (n=4).

Findings are presented as analytic patterns organized within the BeSD domains, with contrasts described when participants differed in their knowledge, attitudes, circumstances, and reported likelihood of accepting the RSV vaccine during their current pregnancy.

### Motivation Toward the Uptake of RSV Immunization

Table 1 presents participants’ self-reported RSV vaccination status at the time of data collection, as well as their willingness to receive the RSV vaccine during their current pregnancy or to have their child receive the antibody shot. Of the 4 participants who expressed uncertainty or negative intentions toward the maternal vaccine, 2 reported being unsure whether they would have their child receive the antibody shot. No one reported that they probably or definitely would not have their child receive the antibody shot.

**Table 1.** Self-reported likelihood of respiratory syncytial virus (RSV) vaccination during pregnancy and infant antibody uptake^a^.

Likelihood of RSV immunization uptake	Participants, n
Maternal RSV vaccine (n=12)	
Already received the vaccine or definitely or probably would receive it during the current pregnancy	8
Unsure whether to receive the vaccine or probably or definitely would not receive it during the current pregnancy	4
Infant RSV antibody shot (n=12)	
Definitely or probably would have their child receive the antibody shot	5
Unsure whether to have their child receive the antibody shot	2
Received or planned to receive RSV vaccine during the current pregnancy	5

a Data were collected from screening survey prior to the interview.

Intentions to receive the RSV vaccine during pregnancy or to have their child receive the antibody shot were influenced by social norms, emotions, and decision-making processes, as described in the sections below. Prior to pregnancy, most participants reported following the routine immunization schedule. Among those who were accepting of vaccines, intentions were clear, with 1 participant having already received the maternal RSV vaccine and another stating the following:

*I get all the vaccines. If you told me to get a vaccine, I get the vaccine.*

In contrast, a small number of participants expressing unsure or negative intentions or behaviors described having previously received annual vaccines but no longer perceiving these vaccines as beneficial.

The strongest motivator for participants with positive intentions or behaviors toward RSV vaccination was knowledge of or direct experience with severe RSV illness, including emergency room visits and hospitalizations. One participant who had worked as an emergency medical technician explained as follows:

*Seeing those little babies when they’re in severe respiratory distress is just, oh, it’s just awful! So, anything to keep that from happening, I’d be willing to do.*

Although some participants described hearing stories or watching online videos of infants in intensive care, those with firsthand experience were vocal about how these experiences solidified their intention to use any available immunization product. One participant, recalling her child’s hospitalization, emphasized as follows:

*When you actually see it in person. It’s like, Oh, my gosh! I don’t want my child to go through that...even if it means them getting a shot.*

When asked about the 2 available products, participants generally expressed a preference for vaccination during pregnancy over the antibody shot. Similar to participants with expressed positive intentions or behaviors toward RSV vaccination, those with unsure or negative intentions toward maternal vaccination were also influenced by accounts of or experiences with adverse RSV outcomes. However, some participants in this group noted that information was not always enough to change their behaviors, but observing others receive immunization without adverse effects could increase their willingness to do so.

### Thinking and Feeling

Because views of the antibody shot were generally favorable, even among participants with unsure or negative intentions or behaviors toward vaccination, we compared participants with positive intentions or behaviors with those with unsure or negative intentions or behaviors toward maternal RSV vaccination, while also examining contextual factors to identify shared and divergent BeSD (Table 2).

**Table 2.** Contextual factors that impact respiratory syncytial virus (RSV) vaccine intention and behavior.

Contextual factors	Positive intention or behavior	Unsure or negative intention or behavior	Data concordance or discordance
Thinking and feeling			
Professional identity	Participants with medical training (ie, nurses and doulas) or access to family with medical training expressed greater confidence in weighing evidence to make immunization decisions.	Participants without clinical training or access to practicing clinicians described needing to conduct more research themselves when they did not have access to or trust in medical professionals.	Both groups described relying on individuals in their social networks with medical training, although this reliance was less pronounced among those with medical education or training themselves.
Personal health philosophies	Participants often framed themselves as resilient but wanted to be vaccinated to avoid complications during pregnancy or delivery.	Participants described preferring “natural” approaches and avoiding unnecessary medicine.	All participants emphasized the importance of safety for themselves and their infants and expressed a strong desire to prevent their infants from contracting RSV; however, their approaches to prevention differed.
Social processes			
Family and community voices	Participants reported having supportive spouses, family members, or clinicians. Some participants described setting boundaries with family members who held different vaccine attitudes and beliefs.	Participants noted family members reinforcing their views, such as family members who were midwives or clinicians sharing negative experiences with vaccines. Conflicting family attitudes and beliefs reinforced caution.	All participants expressed a desire to be part of a community that supported their attitudes and beliefs about vaccination, even if this meant compromising or downplaying their own preferences to maintain harmony with those who hold opposing views.
Community context and vaccine-related cultural norms	Participants referenced strong provaccine norms in their communities (eg, clinic policies requiring vaccination) or described limiting their social circle in antivaccine communities to people who expressed provaccine views.	Participants referenced local stigma associated with opting out of maternal vaccination.	Participants with positive intentions focused more on locating themselves within provaccine communities, whereas those with unsure or negative intentions were exposed to a diversity of attitudes and beliefs about vaccination.
Practical issues			
Clinic integration	Participants described their obstetricians as stocking routine vaccines.	Participants more often described encountering fragmented care or uncertainty about whether their clinician administered RSV vaccines.	Both groups described differences in access based on clinic setup, and challenges obtaining vaccines when vaccination required traveling to another location.
Timing and workload	Participants described clinic reminders and clinician prompts as helpful in nudging them toward immunization.	Participants noted that a lack of proactive communication about immunization reduced their motivation.	The eligibility period of 32 to 36 weeks of gestation presented a practical complication for both groups. All participants wanted to learn about RSV immunization options earlier in pregnancy.

Across groups, vaccination decisions were described as emotionally charged and value-laden, grounded in a desire to protect infants. One participant with positive intentions explained as follows:

*The thought of getting a vaccine while I’m pregnant and giving this baby the antibodies he needs without him necessarily having to get poked from the very start, I love that idea.*

Similarly, a participant uncertain about RSV vaccination emphasized the benefit that “the baby is getting protection before she even is out of my womb,” highlighting the reassurance associated with early protection.

Participants in both groups weighed risks and benefits carefully, often citing disease susceptibility and severity. When asked about concerns regarding adverse effects, 1 participant explained as follows:

*[That’s] not something I’m worried about...RSV, I think [about] the negative outcomes associated with the infections that the vaccines are preventing...And that’s the reason that I want vaccinations.*

Even among those receptive to the antibody shot, many prioritized vaccination during pregnancy because it provided earlier protection. A participant who had experienced a newborn hospitalization stated the following:

*If there was a chance where they said like immediately after birth they could give the RSV [antibody shot] I would have still not agreed for the simple fact that it takes at least a week and a half for the antibodies to get established in his blood and in that week and a half there might be chances where could still get the RSV infection.*

Seasonal RSV risk, particularly in households with school-aged children, also influenced decision-making. All participants with unsure or negative intentions or behaviors leaned toward postnatal protection with the antibody shot.

Participants with positive intentions or behaviors toward RSV vaccination often had backgrounds in science, research, or medicine and reported trusting experts and the health care system. They framed vaccination as evidence-based and aligned with scientific consensus, stating the following:

*Experts have said the risk of these diseases is higher than the risk of the vaccine side effects. So, I’m going to go with their expertise.*

These participants described greater fear of infection than of vaccination and expressed confidence and relief in their decisions.

Participants with unsure or negative intentions toward the maternal RSV vaccine emphasized infant protection but expressed safety concerns, frequently citing the novelty of the vaccine and uncertainty about long-term developmental outcomes. One participant explained as follows:

*With this RSV one being so new...you feel almost like a guinea pig in a way.*

These participants were generally comfortable with the antibody shot, with 1 noting the following:

*It just makes me feel more comfortable... I want to give their body time to just develop before I start giving them medicine.*

Participants with unsure or negative intentions or behaviors often referenced anecdotal experiences of side effects among family members and reported limited knowledge of RSV immunization options. Many expressed a desire for clinicians to initiate conversations and share data, ingredient lists, and real-world experiences. Some described feeling stigmatized when declining vaccines, as 1 participant recalled the following:

*When you do decline a vaccine, there is almost like a stigma against it.*

Attitudes toward other seasonal vaccines, such as influenza vaccines and COVID-19 vaccines, were mixed, with some participants expressing greater confidence in vaccines with longer track records.

### Social Processes

Both groups described vaccination decision-making as a socially embedded process shaped by close relationships and trusted medical professionals. Participants emphasized that both the source and the manner of communication mattered.

Among those with unsure or negative intentions toward RSV vaccination, several preferred direct, personalized guidance from their obstetricians. One participant valued her clinician’s straightforward communication and, as a nonclinician, described relying on colleagues with clinical training for “a synopsis...key takeaways,” noting that she would not independently seek out educational articles when trusted expertise was readily available. Others described clinicians as overly passive when maternal vaccination was discussed neutrally during prenatal visits. As 1 participant explained, “If my [obstetrician] would have pushed it a little more, I probably would have got it,” referring to her decision not to receive other maternal vaccines. These participants sought information grounded in lived experience, often turning to experienced clinicians, peers who had made similar decisions, or parents of children who had had RSV.

In contrast, participants with positive intentions or behaviors toward RSV vaccination also emphasized trusted clinicians but were more willing to independently seek out and evaluate information. They described comparing claims across sources to verify guidance and highlighted partner engagement as central to decision-making. Many also reported access to medically trained relatives, which reinforced their confidence in vaccination decisions. By comparison, those with unsure or negative intentions described social environments characterized by diverse or conflicting vaccination beliefs.

Across groups, participants emphasized the importance of face-to-face communication and providing information early, before the eligibility window opened, allowing time for reflection and discussion with support persons. Figure 1 depicts prenatal care time points at which participants felt clinicians could introduce RSV immunization options and address questions. These time points were identified as participants discussed what would make them feel more comfortable receiving the RSV vaccine during pregnancy, including having information introduced with sufficient time to consider their options and discuss questions with clinicians. Participants noted that information provided through patient portals or text messages was easy to “scroll right through...” Both groups valued having access to written materials, either printed or online, that could be revisited and discussed with partners or clinicians before making a decision.

Opinions about social media varied widely. Some participants viewed it as a helpful source of shared experiences, while others dismissed it as unreliable or harmful. For many participants with unsure or negative intentions toward maternal RSV vaccination, social media was an entry point for information seeking, including online discussion boards presenting multiple perspectives. One participant noted the following:

*I don’t think I’d need to know them personally to receive what they have to say.*

A minority of participants, all with positive vaccine intentions and behaviors, avoided social media entirely when making this decision. Across groups, trust and credibility were central, whether expressed through reliance on physicians, pediatricians, or select websites or through skepticism toward institutions and a preference for peer narratives.

**Figure 1.**
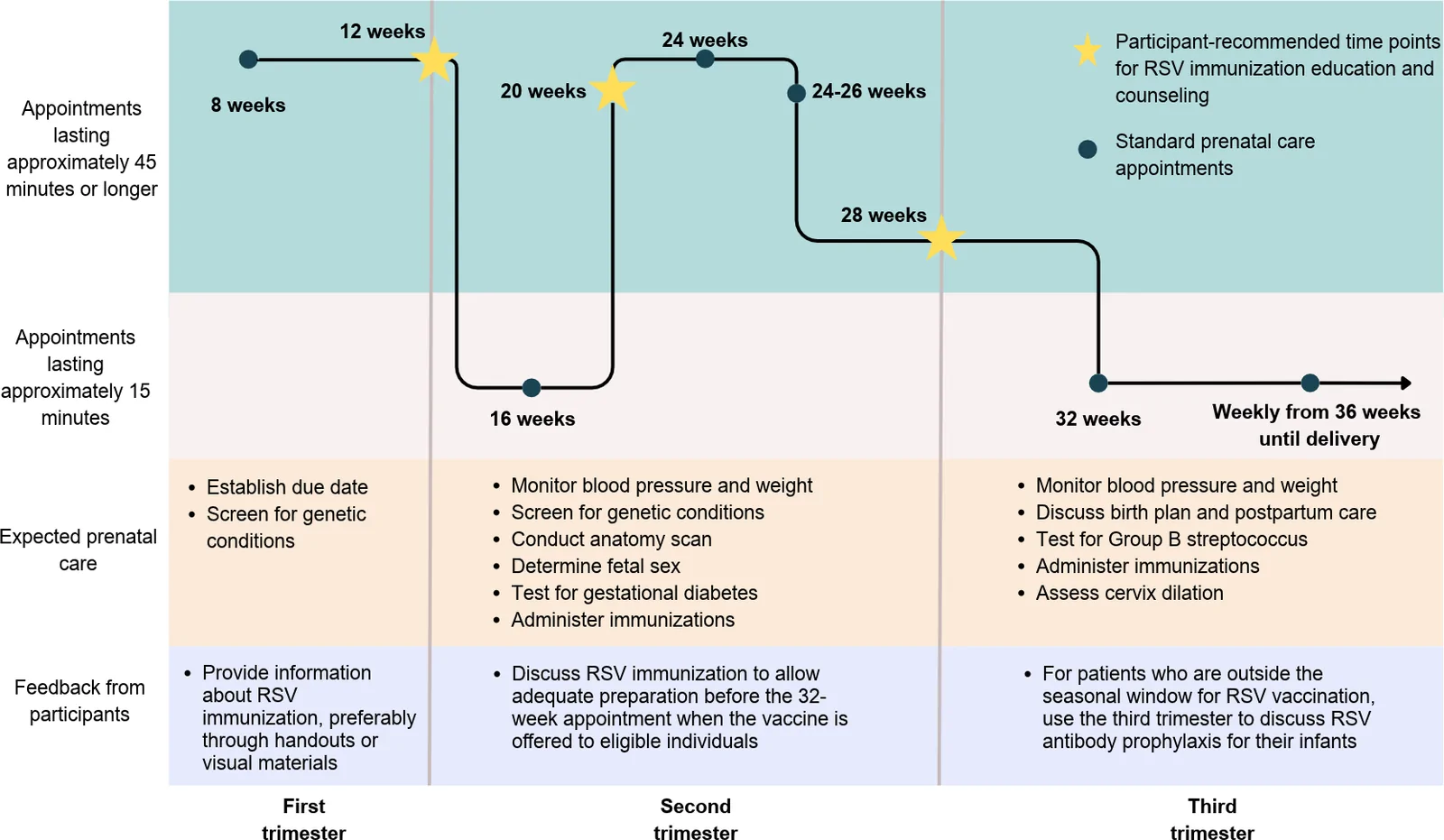
Recommended time points for integrating education and counseling on respiratory syncytial virus (RSV) immunization products among pregnant and postpartum women in the United States (2024-2025). The figure illustrates participants’ recommendations for introducing discussions about RSV immunization across the prenatal care continuum, alongside typical prenatal appointments and routine clinical milestones. Star symbols indicate participant-recommended time points for RSV immunization education. Participants consistently emphasized the importance of introducing information early in pregnancy (approximately 12-20 weeks of gestation), reinforcing discussions during the second trimester, and revisiting maternal RSV immunization at the time of vaccine eligibility (32-36 weeks of gestation). Recommendations also highlighted providing clear educational materials, discussing maternal RSV vaccination before the eligibility window, and counseling individuals who are not vaccinated during pregnancy about infant RSV monoclonal antibody protection after birth. The appointment schedule shown illustrates a standard sequence of prenatal visits for an uncomplicated pregnancy and highlights points where additional resources or supports could be incorporated into routine care.

### Practical Issues

All participants described RSV vaccination as a practical decision shaped by access, timing, and convenience. Vaccination was viewed not only as a matter of belief but also of logistics, including availability in prenatal care settings, scheduling effort, and insurance coverage. Across groups, convenience and clear communication from trusted clinicians strongly influenced follow-through, particularly when eligibility was uncertain.

Participants with unsure or negative intentions toward RSV vaccination often described uncertainty about where to access the vaccine or whether it was offered by their obstetrician. Others cited transportation and scheduling burdens, describing vaccination as requiring effort to “drive to CVS and make the appointment and take the time out of the day to do it.” Some lived in areas with limited clinician availability, with 1 person stating, “I have to drive an hour away to see an [obstetrician],” linking clinician scarcity to missed in-office vaccination opportunities. These participants emphasized that logistical friction, rather than outright opposition, often separated intention from uptake.

In contrast, participants with positive intentions or behaviors toward RSV vaccination reported fewer structural barriers and greater confidence in navigating health care systems. They described proximity to clinics or pharmacies that routinely stocked vaccines and familiarity with receiving vaccines during prenatal visits. Although acknowledging cost as a potential barrier for others, most did not view it as personally prohibitive. One participant explained as follows:

*If I didn’t have insurance, then cost would absolutely be more of a factor...but things being widely available and lower in cost is going to increase uptake.*

These participants valued advance notice about RSV eligibility and found hospital messaging effective in normalizing vaccination. Several preferred vaccination during pregnancy to avoid postpartum scheduling, describing it as “one more thing that’s done ahead of time.”

Across groups, participants emphasized that RSV vaccination should be integrated into routine prenatal care rather than requiring separate appointments or locations.

## Discussion

### Principal Findings

Our findings show that RSV immunization decisions during pregnancy are shaped by practical, social, and relational factors rather than attitudes alone. Participants across intention groups relied on close family members and clinicians as key reference points but differed in how they sought and acted on information. Those with positive vaccination intentions and behaviors more actively evaluated evidence, triangulated sources, and relied on medically informed networks, whereas those with unsure or negative intentions were more influenced by perceived judgment from clinicians and peer experiences. These findings suggest that strengthening clinician recommendations and embedding maternal RSV vaccines within routine prenatal visits may reduce barriers and narrow the gap between vaccine intention and uptake, supporting evidence that many families prefer infant RSV immunization through maternal vaccination [10,18]. Consistent with our objective of examining the BeSD of RSV immunization decisions, these findings demonstrate that decision-making is shaped not only by vaccine confidence but also by the timing, source, and delivery of information within routine prenatal care.

### Comparison to Prior Work

Across participant accounts, we identified convenience, accessibility, and clear communication from trusted clinicians as important influences on vaccination decisions, reinforcing evidence that logistical ease and health care touchpoints are central to vaccination [19,20]. Clinician communication efforts should therefore emphasize not only accurate information but also the delivery style, including empathy, decisiveness, and tailored responses to safety concerns. Engaging supportive networks, including partners and medically trained family members, may further normalize RSV vaccination and help counteract conflicting beliefs. These findings align with prior studies highlighting the importance of clinician recommendations and trust in health care systems, particularly for newer vaccines and within the narrow RSV vaccination eligibility window [10,19-23].

Prior maternal vaccination research has similarly identified vaccine safety concerns, perceived benefits and risks, and health care clinician recommendations as important influences on vaccination decisions during pregnancy [24-26]. Our findings build on this literature by demonstrating that RSV immunization decisions are distinct from those involving traditional maternal vaccination because pregnant individuals must navigate 2 complementary prevention options that differ in timing, delivery, and perceived advantages. Participants generally discussed these approaches as complementary rather than competing options, although maternal vaccination was often preferred because of the perceived benefit of providing protection earlier. This differs from decision-making around more established maternal vaccines, for which an alternative infant-administered preventive option is not typically part of the decision, as it is with RSV immunization [2]. Our interpretation that uncertainty was often related to questions regarding eligibility, logistics, and available information is also consistent with emerging RSV literature describing knowledge gaps and uncertainty surrounding newly introduced maternal and infant RSV prevention options [27]. Together, these findings suggest that RSV education may need to extend beyond addressing vaccine confidence to clearly explain the timing, eligibility, and relative roles of both prevention options.

### Implications

Our findings also have implications for prenatal care delivery. Participants consistently described wanting information before reaching the 32- to 36-week vaccination window, suggesting that RSV immunization discussions should begin earlier in pregnancy and be revisited over multiple prenatal visits rather than occurring as a single recommendation at the time of vaccine eligibility. The patient journey map developed in this study offers a potential framework for integrating education and counseling into routine prenatal care. Future work should evaluate patient-centered educational materials introduced earlier in pregnancy alongside implementation strategies that strengthen clinician communication, integrate RSV counseling into prenatal workflows, and reduce logistical barriers to maternal vaccination. These strategies are collectively needed to support informed decision-making that responds to documented information needs and vaccine intentions among pregnant individuals [28-31]. Longitudinal and implementation-focused studies are also needed to assess how educational tools, clinician communication approaches (eg, strong recommendation framing, shared decision-making, or partner-inclusive counseling), and strategies to reduce logistical barriers influence decision-making across pregnancy and the postpartum period, particularly among first-time parents who report greater uncertainty about childhood vaccinations [29,32]. As maternal RSV vaccines expand globally [33], implementation strategies and patient-centered interventions that are responsive to interpersonal, organizational, and structural influences will be critical for improving uptake and protecting infants from severe RSV outcomes.

### Strengths and Limitations

This qualitative study enabled in-depth exploration of immunization intentions and decision-making, providing rich contextual insight. Remote interviewing and purposive sampling ensured the inclusion of participants with positive and unsure or negative intentions toward RSV immunization products. Several limitations warrant consideration. Screening questions assessing willingness to use the RSV antibody shot did not capture whether participants who received or planned to receive the maternal vaccine would consider the antibody shot if they were ineligible for maternal vaccination during pregnancy. Instead, this information was explored during interviews. Some participants may have reported negative vaccine intentions because their anticipated delivery dates fell outside the vaccination eligibility window, although no concerns about eligibility were raised during staff-administered screening, and few participants demonstrated awareness of vaccination timing recommendations. One participant expressed mixed responses across screening items and was classified as unsure based on vaccine intention. In addition, interviews were conducted during and immediately following the RSV season, a period when media coverage, health care messaging, and personal experiences with RSV may have heightened awareness of the disease and influenced participants’ perceptions of RSV risk and immunization. Although parity data were not collected, participants were encouraged to discuss prior pregnancy experiences when describing their RSV immunization decisions. Consequently, we were unable to compare decision-making between first-time and experienced parents. Finally, the sample was relatively homogeneous, consisting primarily of White, English-speaking adults from the southern United States, with education or employment in public health or medicine. Recruitment through ResearchMatch, health care settings, and community-based outreach may have influenced the composition of the sample by preferentially reaching individuals who were more engaged with health care or research and may have contributed to the geographic concentration of participants from the southern United States. Although purposive sampling successfully captured participants with varying RSV immunization intentions, these characteristics may limit the transferability of the findings to pregnant populations with different demographic, geographic, educational, and health care experiences. Future maternal vaccine research should intentionally recruit more diverse populations to better understand how these factors shape immunization decision-making across pregnancy.

### Conclusions

RSV immunization decisions during pregnancy are shaped by interpersonal trust, logistical access, and the quality and timing of clinician communication. As maternal RSV immunization programs continue to expand, successful implementation will depend not only on vaccine availability but also on integrating timely education, trusted counseling, and streamlined clinical workflows into routine prenatal care to support informed decision-making and improve infant protection.

## Supplementary material

10.2196/100656 Multimedia Appendix 1 Interview guide.

## Abbreviations

BeSD: behavioral and social drivers
COREQ: Consolidated Criteria for Reporting Qualitative Research
RSV: respiratory syncytial virus
WHO: World Health Organization
