# Supplementary material for: Decision-Making About Respiratory Syncytial Virus Immunization for Infants: Qualitative Interview Study
Source: JMIR Form Res. 2026 Nov 9;10:e100656. doi: 10.2196/100656 (PMC13747271; doi:10.2196/100656)
Supplement: Supplementary file 1 [file formative-v10-e100656-s001.pdf]

Maternal Vaccination Study In-Depth Interview Guide

1) Welcome, consent process, and introduction

2) Interview Questions

Warm Up

1. To start, who do you trust the most to support you in making decisions about your health during pregnancy and why?

Thinking and Feeling

During pregnancy there are many decisions you must make that may impact the health of you and your baby. Among those is the decision to get vaccinated.

2. How do you feel about getting vaccinated in pregnancy?

Currently, it is recommended that pregnant people get four vaccines during pregnancy. These vaccines are the Tetanus-Diphtheria-acellular Pertussis (Tdap) vaccine, the flu vaccine, the COVID-19 vaccine, and a new vaccine against respiratory syncytial virus (RSV).

3. What have you heard about these vaccines recommended during pregnancy (Flu, Tdap, COVID-19, RSV)? *(Probes: Types of sources (e.g., family, healthcare providers, religious leaders); heard anything concerning/believe that it is true. Do you work in a field related to public health or medicine?)*

Motivation

4. What are the main reasons you would want to get vaccinated during pregnancy (Probes: Your community/people you know. Does this differ from other people you know that are pregnant?)?

5. What are the main reasons you would not want to get vaccinated during pregnancy (*Probes: (mis)information, attitudes towards vaccines, fear of side effects, trust in medical system, lack of social support. Does this differ from other people you know that are pregnant?)?*

RSV Specific

Having discussed some of the reasons why pregnant people may or may not want to get vaccinated during pregnancy, I want us now to focus specifically on the RSV vaccine products for the next few questions.

Respiratory syncytial (sin-SISH-uhl) virus, or RSV, is a common respiratory virus that usually causes mild, cold-like symptoms such as cough, runny nose, wheezing, and fever. Most people recover in a week or two, but RSV can be serious, especially for infants and older adults. RSV is the most common cause of bronchiolitis (inflammation of the small airways in the lung), pneumonia (infection of the lungs), and hospitalization in children younger than 1 year of age in the United States.

There are currently two ways to protect children from RSV infection: Vaccinating the mother during pregnancy, or giving an antibody shot to the child between birth and 8 months of age.

Antibody shot for child: Antibodies are proteins that are made by your body when it fights against an infection and help prevent infection from attacking the body. Children who are too young to make antibodies against RSV themselves may be able to get RSV antibodies (e.g., as a shot) to protect them from RSV infection. An FDA-approved, CDC-recommended RSV antibody shot called nirsevimab (brand name: Beyfortus) is available and recommended for children younger than 8 months of age during RSV season (fall/winter/spring).

Vaccination during pregnancy: Children can also receive RSV antibodies from their mother, if the mother got vaccinated against RSV during pregnancy. An FDA-approved, CDC-recommended RSV vaccine (Pfizer's Abrysvo) is available and recommended for pregnant people during weeks 32 through 36 of pregnancy from September through January. Vaccination in pregnancy protects children from RSV infection after birth.

6. Based on your survey responses, you (have/**have not**) received your RSV vaccine during pregnancy-is that correct**? And you indicated you will likely not receive the vaccine, but may consider the antibody shot**
    - ii. If yes: **what led you to make that decision?** (If yes, skip questions 7 and 8.)
    - iii. If not (and has delivered): has your child received the antibody shot?

If no:

7. What information would you need to feel comfortable getting the RSV vaccine during pregnancy? (what does credible information source look like to you?)

8. What information would you need to feel comfortable getting the RSV antibody shot for their child after birth?

(*Probe: difference in factors impacting the decision for an antibody shot vs maternal vaccination)*

Social Processes

Thank you for sharing your thoughts on what drives people's decisions to get vaccines during pregnancy. I would now like to shift to discussing the sources of information people rely on when making those decisions.

9. What types of information sources do you look to when making vaccination decisions? (*Probe: assessing credibility, relationship to information source, timing of message)*

10. Can you describe a time when someone has spoken to you about vaccinating during pregnancy? (Probes: types of people, influence, positive/negative, mode of communication, types of information received, when in pregnancy, if no discussions—preference for having them)

11. (If vaccinated) What did the person say to you that influenced you to get vaccinated?

Conclusion--Practical Issues

As we come to the end of our discussion, I have one final question.

12. How easy do you think it is for pregnant people in your community to get a vaccine if they wanted to? *(Probes: Access to clinics; affordability; availability of vaccines; scheduling conflicts)*

Is there anything we did not cover in our discussion today that you would like to mention at this time?

Thank you for taking the time to share your opinions. We appreciate your time and input!
